# Possible Pharmacological Approach Targeting Endoplasmic Reticulum Stress to Ameliorate Leptin Resistance in Obesity

**DOI:** 10.3389/fendo.2016.00059

**Published:** 2016-06-08

**Authors:** Toru Hosoi, Koichiro Ozawa

**Affiliations:** ^1^Department of Pharmacotherapy, Graduate School of Biomedical and Health Sciences, Hiroshima University, Hiroshima, Japan

**Keywords:** endoplasmic reticulum stress, obesity, leptin, leptin resistance, STAT3

## Abstract

Obesity is associated with metabolic syndrome, such as diabetes, hypertension, and hyperlipidemia. Therefore, drug development for the treatment of obesity is needed. Leptin is an anti-obesity hormone that inhibits food intake and increases energy metabolism, and, as such, treatments involving leptin were expected to be beneficial for obesity; however, since most obese patients are in a state of leptin resistance, these treatments may not be useful. Therefore, the amelioration of leptin resistance has recently been attracting interest as a treatment for obesity. The mechanisms underlying the development of leptin resistance need to be elucidated in more detail. Endoplasmic reticulum (ER) stress was recently suggested to be involved in the pathogenesis of leptin resistance. The molecular mechanisms responsible for leptin resistance and possible pharmacological treatments for obesity have been discussed herein, with a focus on ER stress.

## Leptin

Leptin is an anti-obesity hormone that was first discovered by Dr. Friedman’s group in the year 1994 (1). It is secreted from adipose tissue and circulates in the bloodstream. Several splicing isoforms of the leptin receptor have been identified to date, such as Ob-Ra, Ob-Rb, Ob-Rc, Ob-Rd, Ob-Re, and Ob-Rf. Of these, the Ob-Rb isoform is the longest and has Box 1, 2, and 3 regions, which are important for Janus kinase 2 (JAK2) and signal transducer and activator of transcription 3 (STAT3) binding. Upon binding to the Ob-Rb receptor, leptin activates JAK2/STAT3 signaling. The Ob-Rb long isoform is mainly expressed in the hypothalamus (2, 3). In addition to the hypothalamus, we also previously demonstrated that a functional leptin receptor of Ob-Rb is expressed in the brain stem (4). We reported that leptin has the ability to activate STAT3 signaling in the hypothalamus and the brain stem (4). Since a mutation in the Ob-Rb receptor in db/db mice results in severe obesity, the Ob-Rb receptor is considered to play an important role in the anti-obesity effects of leptin. Furthermore, the importance of Ob-Rb–STAT3 signals from leptin receptors was demonstrated by the replacement of Tyr 1138 in Ob-Rb with a serine residue, which specifically disrupted Ob-Rb–STAT3 signaling in mice, resulting in obesity (5). Other isoforms, such as Ob-Ra, Ob-Rc, Ob-Rd, and Ob-Rf, are the short isoforms of leptin receptors and only have the Box 1 region. These isoforms activate JAK2, but not STAT3 signaling, and, as such, are not involved in the anti-obesity effects of leptin. We previously reported that these short isoforms may play a role in regulating immune function through the induction of interleukin (IL)-1β (6) and IL-1 receptor antagonists (7). However, the physiological roles of the short isoforms of leptin receptors have yet to be investigated in detail. On the other hand, the Ob-Re isoform of the leptin receptor is known to be a soluble type that does not have a transmembrane region, and it circulates in the bloodstream and regulates leptin concentrations (8).

## Leptin Resistance

**Figure 1:**
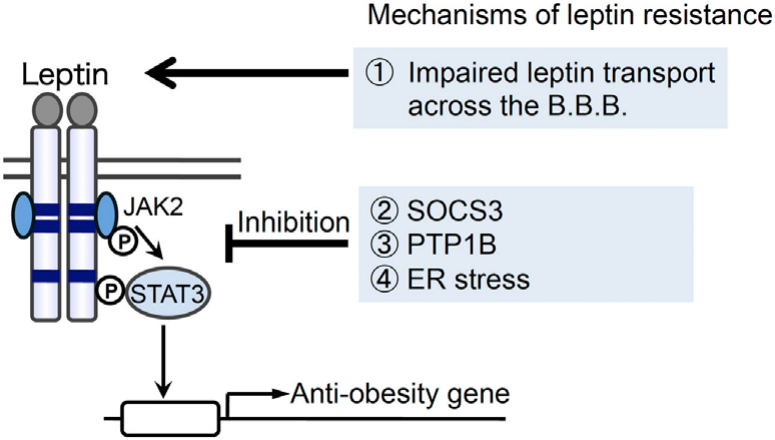
**Possible mechanisms for the development of leptin resistance with obesity**. Several mechanisms have been suggested for the development of leptin resistance: (1) impaired leptin transport across the blood–brain barrier (BBB), (2) the induction of suppressor of cytokine signaling 3 (SOCS3), (3) the induction/activation of protein tyrosine phosphatase 1B (PTP1B), and (4) endoplasmic reticulum (ER) stress have been suggested to play roles in the development of leptin resistance in obesity.

The discovery of leptin led to the expectation that leptin therapy may be beneficial for obesity. However, since circulating levels of leptin are elevated with obesity (9), and most obese patients are in a state of leptin resistance, leptin therapy may not be beneficial for most obese patients (10). Therefore, a clearer understanding of the mechanisms underlying the development of leptin resistance is considered as important. One of the mechanisms suggested to be responsible for leptin resistance is the impaired transport of leptin across the blood–brain barrier (BBB) (11, 12) (Figure 1). A previous study showed a decrease in the ratio of leptin levels in cerebrospinal fluid versus serum with obesity, suggesting that the capacity for leptin transport is lower in obese patients (13). Furthermore, impairments in leptin-induced STAT3 phosphorylation were demonstrated in a mouse model of diet-induced obesity following a peripheral, but not central injection of leptin (14).

Another possibility for the development of leptin resistance is impaired intracellular signal transduction. Previous studies reported that negative feedback regulators of JAK2/STAT3 signal transduction increase with obesity. Suppressor of cytokine signaling 3 (SOCS3) is a negative feedback regulator (15–18) that acts downstream of the leptin receptor (19, 20) (Figure 1). The expression of SOCS3 was previously shown to be regulated through STAT3 and negatively regulates the effects of leptin (19, 20). In addition to SOCS3, protein tyrosine phosphatase 1B (PTP1B) also plays a role in inhibiting the effects of leptin by de-phosphorylating JAK2 (21–23) (Figure 1). Furthermore, previous studies demonstrated that SOCS3 and PTP1B levels were elevated in a mouse model of obesity (19, 24, 25), suggesting the involvement of SOCS3 and PTP1B in leptin resistance with obesity.

The endoplasmic reticulum (ER) is an organelle that is involved in promoting protein folding (26–28). Various stresses, which impair ER function, lead to the accumulation of unfolded proteins in the ER and cause ER stress. ER stress has been implicated in several types of diseases, such as neurodegenerative disease, diabetes, and obesity (18, 28). Cells activate unfolded protein responses (UPR), such as double-stranded RNA-activated protein kinase (PKR)-like ER kinase (PERK)-eukaryotic initiation factor 2 (eIF2α), inositol-requiring enzyme-1 (IRE1)-X-box-binding protein 1 (XBP1), and activating transcription factor 6 (ATF6) pathways, upon the accumulation of unfolded proteins (26–28). Recent findings suggest that ER stress is also involved in the development of leptin resistance (Figure 1). Previous studies demonstrated that ER stress was enhanced in a mouse model of obesity (29) and was suggested to be involved in the development of leptin resistance (30–32). One of the physiological factors that may cause ER stress is saturated fatty acids. Palmitate, a saturated fatty acid, but not oleate, was shown to increase ER stress in the pancreatic β cell line, INS-1 (33). Therefore, the excessive intake of saturated fatty acids may cause ER stress and leptin resistance in obesity. Furthermore, homocysteine has been reported to cause ER stress (34, 35). We previously demonstrated that peripherally injected homocysteine induced XBP-1 splicing in the mouse brain (36), and homocysteine inhibited leptin-induced STAT3 phosphorylation in the mouse hypothalamus (30). Since plasma homocysteine levels increase with obesity (37), homocysteine-induced ER stress may also play a role in the development of leptin resistance.

## Possible Pharmacological Approach to Ameliorate Leptin Resistance by Targeting ER Stress

As discussed above, leptin resistance is associated with obesity, and, thus, its amelioration may represent an important therapeutic approach for obese patients. One of the potential pharmacological approaches for obesity is the use of ER stress modulators. In this review, we focused on the attenuation of ER stress, a recently suggested pharmacological strategy to ameliorate leptin resistance.

Several compounds have been reported to ameliorate ER stress and inhibit obesity. For example, fluvoxamine, a selective serotonin reuptake inhibitor, was previously shown to reduce leptin resistance in neuronal cells (38). Fluvoxamine has affinity for the sigma-1 receptor (Sig-1R) (39), which is an ER protein involved in ER stress (40). Since fluvoxamine has the ability to reduce ER stress through Sig-1R (41), its pharmacological effect of attenuating leptin resistance may be due to a reduction in ER stress (38). In addition to fluvoxamine, another compound attracting interest is celastrol, which was identified in the roots of *Tripterygium wilfordii* (thunder god vine) (42). It was found to ameliorate hypothalamic ER stress and the development of obesity in mouse models (42).

Chemical chaperones are chemical compounds that assist in protein folding (43) and have been suggested to ameliorate ER stress-induced leptin resistance. For example, the chemical chaperone, 4-phenyl butyric acid (4-PBA), was previously reported to ameliorate ER stress-induced leptin resistance (30, 31). Furthermore, the non-steroidal anti-inflammatory drug (NSAID), flurbiprofen, was shown to function as a chemical chaperone and ameliorate ER stress-induced leptin resistance and high fat diet-induced obesity (44). Caffeine also exhibited chaperone activity and reduced ER stress-induced leptin resistance in a neuronal cultured cellular model (45).

In addition to a strategy to ameliorate ER stress through the use of chemical chaperones, pharmacological modulators of UPR may be beneficial. The activation of UPR may induce protein folding signals, which then inhibit the accumulation of unfolded proteins in the ER. The activation of UPR by the overexpression of an ER stress sensor protein has been shown to attenuate ER stress-induced leptin resistance (31). Moreover, the expression of the spliced form of XBP1 in pro-opiomelanocortin (POMC) neurons was found to improve ER stress-induced leptin insensitivity (46). Therefore, future studies to identify and evaluate the anti-obesity effects of these types of compounds, which have the ability to modulate the effects of UPR, will be of interest. On the other hand, since the activation of UPR was suggested to be involved in cancer development (47), potential side effects must also be carefully considered when developing drugs to modulate UPR.

Overall, targeting ER stress may be beneficial for the treatment of obesity. This approach may assist in enhancing the effects of endogenous leptin on leptin resistance in obese patients. The identification and elucidation of the pharmacological mechanisms of action of drugs targeting obesity warrant further study.

## Author Contributions

TH wrote the manuscript. KO checked the manuscript.

## Conflict of Interest Statement

The authors declare that the research was conducted in the absence of any commercial or financial relationships that could be construed as a potential conflict of interest.
